# Hydroxonium 1-ammonio­ethyl­idene-1,1-bis­phospho­nate

**DOI:** 10.1107/S1600536808029565

**Published:** 2008-09-20

**Authors:** Ying Wu, Du-Lin Yin

**Affiliations:** a Yueyang Vocational Technology College, Yueyang 414000, Hunan, People’s Republic of China; b College of Chemistry and Chemical Engineering, Hunan Normal University, Changsha 410017, Hunan, People’s Republic of China

## Abstract

The title compound, H_3_O^+^·C_2_H_8_NO_6_P_2_^−^, contains a disordered H_3_O^+^ cation and an NH_3_C(CH_3_)(PO_3_H)_2_ anion. The three H atoms of the H_3_O^+^ cation are statistically distributed over four positions with occupancies of 0.75, resulting in a pseudo tetra­hedron. Multiple N—H⋯O and O—H⋯O hydrogen bonds generate an intricate three-dimensional network.

## Related literature

For related literature, see: Bollinger & Roundhill (1993 ▶); Chai *et al.* (1980 ▶); Clearfield (2002 ▶); Fernández *et al.* (2003 ▶); Li *et al.* (2008 ▶); Finn *et al.* (2003 ▶); Yin *et al.* (2005 ▶). 
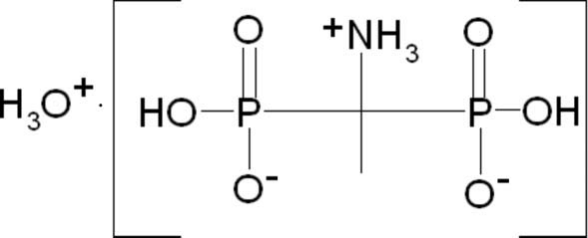


## Experimental

### 

#### Crystal data

H_3_O^+^·C_2_H_8_NO_6_P_2_^−^
*M* *_r_* = 223.06
Triclinic, 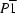

*a* = 5.6379 (5) Å
*b* = 8.9712 (8) Å
*c* = 9.2302 (8) Å
α = 102.111 (1)°
β = 100.499 (1)°
γ = 101.342 (1)°
*V* = 435.22 (7) Å^3^
*Z* = 2
Mo *K*α radiation
μ = 0.50 mm^−1^
*T* = 293 (2) K
0.36 × 0.32 × 0.27 mm

#### Data collection

Bruker SMART 4K CCD area-detector diffractometer
Absorption correction: multi-scan (*SADABS*; Sheldrick, 1996 ▶) *T*_min_ = 0.839, *T*_max_ = 0.876
2811 measured reflections
1946 independent reflections
1871 reflections with *I* > 2σ(*I*)
*R*_int_ = 0.009

#### Refinement

*R*[*F* ^2^ > 2σ(*F* ^2^)] = 0.034
*wR*(*F* ^2^) = 0.095
*S* = 1.08
1946 reflections
113 parameters
H-atom parameters constrained
Δρ_max_ = 0.44 e Å^−3^
Δρ_min_ = −0.58 e Å^−3^

### 

Data collection: *SMART* (Bruker, 2001 ▶); cell refinement: *SAINT* (Bruker, 2001 ▶); data reduction: *SAINT*; program(s) used to solve structure: *SHELXS97* (Sheldrick, 2008 ▶); program(s) used to refine structure: *SHELXL97* (Sheldrick, 2008 ▶); molecular graphics: *ORTEP-3 for Windows* (Farrugia, 1997 ▶) and *PLATON* (Spek, 2003 ▶); software used to prepare material for publication: *SHELXL97*.

## Supplementary Material

Crystal structure: contains datablocks I, global. DOI: 10.1107/S1600536808029565/pv2098sup1.cif

Structure factors: contains datablocks I. DOI: 10.1107/S1600536808029565/pv2098Isup2.hkl

Additional supplementary materials:  crystallographic information; 3D view; checkCIF report

## Figures and Tables

**Table 1:** Hydrogen-bond geometry (Å, °)

*D*—H⋯*A*	*D*—H	H⋯*A*	*D*⋯*A*	*D*—H⋯*A*
N1—H1*A*⋯O3^i^	0.89	2.02	2.798 (2)	145
N1—H1*B*⋯O2^ii^	0.89	2.00	2.766 (2)	144
N1—H1*C*⋯O6^i^	0.89	1.93	2.771 (2)	156
O1—H1⋯O3^iii^	0.82	1.69	2.501 (2)	168
O4—H4⋯O2^iv^	0.82	1.84	2.635 (2)	164
O1*W*—H9⋯O1^v^	0.89	2.06	2.923 (2)	164
O1*W*—H10⋯O5	0.89	1.88	2.737 (2)	164
O1*W*—H11⋯O6^i^	0.88	1.94	2.781 (2)	159
O1*W*—H12⋯O5^vi^	0.89	2.01	2.901 (3)	180

Symmetry codes: (i) 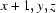; (ii) 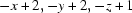; (iii) 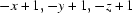; (iv) 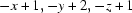; (v) 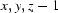; (vi) 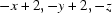.

**Fig. 1.**
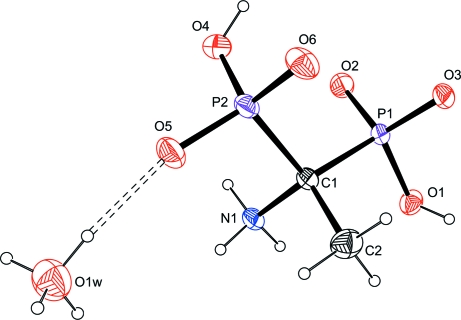
The asymmetric unit of (I) with the atom-labelling scheme. Displacement ellipsoids are drawn at the 30% probability level. H atoms are represented as small spheres of arbitrary radii. Hydrogen bond is shown as dashed line.

**Fig. 2.**
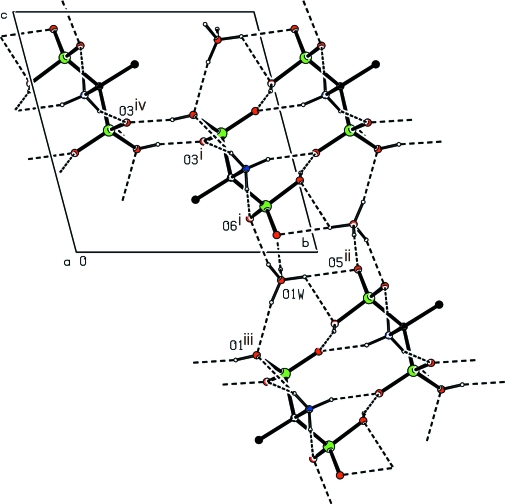
Partial packing view of compound ( I ), showing the formation of the three dimensional network built from hydrogen bonds. For clarity, H atoms not involved in hydrogen bonding have been omitted. [symmetry codes: (i) 1+x, y, z; (ii) 2-x , 2-y, z ; (iii) x, y , z-1; (iv) 1-x, 1-y, 1-z].

## supplementary crystallographic information

### Comment

Phosphonic acids are interesting ligands. They can complex various metal ions and a series of organic-inorganic hydrid materials containing phosphonic acids have been prepared and characterized. Such materials have potential applications in catalysts, sensors, sorbents, magnetic and luminescent materials (Finn *et al.*, 2003). In addition, introduction of some functional groups to phosphonic acids, such as crown ether, –COOH, –OH, –N*R*_2_ or mixed group will modify their complexing ability (Clearfield, 2002). 1-Aminoethylidene-1,1-diphosphonic acid (AEDPH_4_) exists as a zwitterion and is inclined to transfer one proton to the amino group (Bollinger & Roundhill, 1993; Fernández *et al.*, 2003; Li *et al.*, 2008 ). Its deprotonation would result in predictable hydrogen aggregates from stronger P—O—H···O—P to weaker C—H···O hydrogen bonds. However, its crystal structure is still unknown (Yin *et al.*, 2005). Herein, we report the structure of the title compound, (I).

The asymetric unit of (I) is built up from one deprotonated AEDPH_4_ anion and a disordered H_3_O^+^ cation which are linked through OW—H···O hydrogen bonds (Fig. 1, Table 1). Two of the four protons of phosphonates are used in the protonation of one for the amino group, the other for H_3_O^+^ cation. The deprotonated AEDPH_3_^-^ anions form two-dimensional (2D) H-bonded layer along the *bc*-plane. The strongest H-bond is O1—H1···O3^iii^ (with O1···O3 distance 2.501 (2)Å), which links the AEDPH_3_^-^ anions into dimers which form an infinite chain along the *b* axis by hydrogen bond O4—H4···O2^iv^ (O4···O2 distance 2.635 (2) Å). Furthermore, three N-H···O H-bonds connect these chains to obtain a 2D layer. The H_3_O^+^ anions bond to the adjacent layers with five Ow—H···O bonds and stabilize the structure. The occurence of different hydrogen bond interactions, N—H···O, O—H···O and OW—H···O results in the formation of an intricated three dimensional network (Fig. 2, Table 1).

### Experimental

The title compound was synthesized according to the US Patent 4239695 (Chai *et al.*, 1980). It was crystallized unexpectedly when 4,4_'_-bipyridine was adding into the AEDPH_4_ H_2_O solution to synthesize the complex. However, the 4,4_'_-bipyridine was not present in the final product.

### Refinement

All H atoms attached to C atoms, N atom and O(hydroxyl) atoms were fixed geometrically and treated as riding with C—H = 0.96 Å (C), N—H = 0.86 Å and O—H= 0.82Å with U_iso_(H) = 1.5U_eq_(C,N or O). H atoms of the H_3_O^+^ cation were located in difference Fourier maps and included in the subsequent refinement using restraints (O-H = 0.86 (1)Å) with U_iso_(H) = 1.5U_eq_(O); in the final stages of refinement their coordinates were fixed. The three hydrogen atoms of the H_3_O^+^ cation are statistically distributed over four positions with occupation factor of 0.75, resulting in a pseudo tetrahedron.

### Crystal data

**Table:**

H_3_O^+^·C_2_H_8_NO_6_P_2_^−^	*Z* = 2
*M**_r_* = 223.06	*F*(000) = 232
Triclinic, *P*1	*D*_x_ = 1.702 Mg m^−^^3^
Hall symbol: -P 1	Mo *K*α radiation, λ = 0.71073 Å
*a* = 5.6379 (5) Å	Cell parameters from 2523 reflections
*b* = 8.9712 (8) Å	θ = 2.4–29.6°
*c* = 9.2302 (8) Å	µ = 0.50 mm^−^^1^
α = 102.111 (1)°	*T* = 293 K
β = 100.499 (1)°	Plate, colorless
γ = 101.342 (1)°	0.36 × 0.32 × 0.27 mm
*V* = 435.22 (7) Å^3^	

### Data collection

**Table:**

Bruker SMART 4K CCD area-detector diffractometer	1871 reflections with *I* > 2σ(*I*)
graphite	*R*_int_ = 0.009
φ and ω scans	θ_max_ = 27.5°, θ_min_ = 2.3°
Absorption correction: multi-scan (SADABS; Sheldrick, 1996)	*h* = −7→7
*T*_min_ = 0.839, *T*_max_ = 0.876	*k* = −9→11
2811 measured reflections	*l* = −11→8
1946 independent reflections	

### Refinement

**Table:**

Refinement on *F*^2^	Primary atom site location: structure-invariant direct methods
Least-squares matrix: full	Secondary atom site location: difference Fourier map
*R*[*F*^2^ > 2σ(*F*^2^)] = 0.034	Hydrogen site location: inferred from neighbouring sites
*wR*(*F*^2^) = 0.095	H-atom parameters constrained
*S* = 1.08	*w* = 1/[σ^2^(*F*_o_^2^) + (0.0451*P*)^2^ + 0.5124*P*] where *P* = (*F*_o_^2^ + 2*F*_c_^2^)/3
1946 reflections	(Δ/σ)_max_ < 0.001
113 parameters	Δρ_max_ = 0.44 e Å^−^^3^
0 restraints	Δρ_min_ = −0.58 e Å^−^^3^

### Special details

**Table:**

Geometry. All esds (except the esd in the dihedral angle between two l.s. planes) are estimated using the full covariance matrix. The cell esds are taken into account individually in the estimation of esds in distances, angles and torsion angles; correlations between esds in cell parameters are only used when they are defined by crystal symmetry. An approximate (isotropic) treatment of cell esds is used for estimating esds involving l.s. planes.

### Fractional atomic coordinates and isotropic or equivalent isotropic displacement parameters (Å^2^)

**Table:**

	*x*	*y*	*z*	*U*_iso_*/*U*_eq_	Occ. (<1)
P1	0.64618 (8)	0.73089 (5)	0.49332 (5)	0.01519 (14)	
P2	0.58222 (8)	0.83546 (6)	0.19085 (5)	0.01897 (14)	
C1	0.7352 (3)	0.7236 (2)	0.3101 (2)	0.0159 (3)	
C2	0.6859 (4)	0.5520 (2)	0.2183 (2)	0.0263 (4)	
H2A	0.7448	0.5493	0.1268	0.039*	
H2B	0.5106	0.5046	0.1924	0.039*	
H2C	0.7717	0.4950	0.2784	0.039*	
N1	1.0110 (3)	0.79403 (19)	0.34632 (18)	0.0177 (3)	
H1A	1.0884	0.7500	0.4126	0.027*	
H1B	1.0421	0.8971	0.3866	0.027*	
H1C	1.0657	0.7766	0.2612	0.027*	
O1	0.8136 (2)	0.63586 (16)	0.57120 (16)	0.0207 (3)	
H1	0.7371	0.5435	0.5513	0.031*	
O2	0.7185 (3)	0.89740 (16)	0.58704 (16)	0.0234 (3)	
O3	0.3743 (2)	0.64928 (16)	0.46080 (17)	0.0218 (3)	
O4	0.6364 (3)	1.00594 (17)	0.29828 (18)	0.0267 (3)	
H4	0.5123	1.0175	0.3295	0.040*	
O5	0.7259 (3)	0.8501 (2)	0.07158 (17)	0.0295 (3)	
O6	0.3106 (3)	0.75734 (19)	0.14090 (16)	0.0262 (3)	
O1W	1.0469 (3)	0.8192 (2)	−0.1160 (2)	0.0403 (4)	
H9	1.0002	0.7549	−0.2088	0.061*	0.75
H10	0.9220	0.8286	−0.0718	0.061*	0.75
H11	1.1434	0.7837	−0.0515	0.061*	0.75
H12	1.1157	0.9206	−0.1025	0.061*	0.75

### Atomic displacement parameters (Å^2^)

**Table:**

	*U*^11^	*U*^22^	*U*^33^	*U*^12^	*U*^13^	*U*^23^
P1	0.0131 (2)	0.0149 (2)	0.0193 (2)	0.00319 (17)	0.00587 (17)	0.00642 (17)
P2	0.0151 (2)	0.0268 (3)	0.0203 (3)	0.00901 (19)	0.00713 (18)	0.01124 (19)
C1	0.0124 (7)	0.0172 (8)	0.0194 (8)	0.0038 (6)	0.0051 (6)	0.0057 (6)
C2	0.0297 (10)	0.0204 (9)	0.0257 (10)	0.0056 (8)	0.0055 (8)	0.0005 (7)
N1	0.0128 (7)	0.0209 (7)	0.0223 (8)	0.0054 (6)	0.0068 (6)	0.0083 (6)
O1	0.0173 (6)	0.0195 (6)	0.0253 (7)	0.0040 (5)	0.0017 (5)	0.0094 (5)
O2	0.0265 (7)	0.0170 (7)	0.0275 (7)	0.0043 (5)	0.0119 (6)	0.0033 (5)
O3	0.0132 (6)	0.0226 (7)	0.0334 (8)	0.0043 (5)	0.0073 (5)	0.0138 (6)
O4	0.0239 (7)	0.0241 (7)	0.0376 (8)	0.0104 (6)	0.0125 (6)	0.0106 (6)
O5	0.0297 (8)	0.0425 (9)	0.0283 (8)	0.0162 (7)	0.0175 (6)	0.0184 (7)
O6	0.0162 (7)	0.0401 (8)	0.0239 (7)	0.0087 (6)	0.0033 (5)	0.0112 (6)
O1W	0.0390 (9)	0.0496 (11)	0.0344 (9)	0.0119 (8)	0.0101 (7)	0.0127 (8)

### Geometric parameters (Å, °)

**Table:**

P1—O2	1.4952 (14)	C2—H2B	0.9600
P1—O3	1.5081 (13)	C2—H2C	0.9600
P1—O1	1.5686 (14)	N1—H1A	0.8900
P1—C1	1.8417 (18)	N1—H1B	0.8900
P2—O5	1.4928 (14)	N1—H1C	0.8900
P2—O6	1.4947 (14)	O1—H1	0.8200
P2—O4	1.5765 (15)	O4—H4	0.8200
P2—C1	1.8497 (19)	O1W—H9	0.8869
C1—N1	1.505 (2)	O1W—H10	0.8852
C1—C2	1.537 (2)	O1W—H11	0.8841
C2—H2A	0.9600	O1W—H12	0.8888
			
O2—P1—O3	115.72 (8)	C1—C2—H2B	109.5
O2—P1—O1	108.61 (8)	H2A—C2—H2B	109.5
O3—P1—O1	111.06 (8)	C1—C2—H2C	109.5
O2—P1—C1	109.30 (8)	H2A—C2—H2C	109.5
O3—P1—C1	108.25 (8)	H2B—C2—H2C	109.5
O1—P1—C1	103.15 (8)	C1—N1—H1A	109.5
O5—P2—O6	118.42 (9)	C1—N1—H1B	109.5
O5—P2—O4	106.13 (9)	H1A—N1—H1B	109.5
O6—P2—O4	112.40 (8)	C1—N1—H1C	109.5
O5—P2—C1	105.96 (8)	H1A—N1—H1C	109.5
O6—P2—C1	108.21 (8)	H1B—N1—H1C	109.5
O4—P2—C1	104.72 (8)	P1—O1—H1	109.5
N1—C1—C2	107.76 (14)	P2—O4—H4	109.5
N1—C1—P1	106.97 (11)	H9—O1W—H10	113.6
C2—C1—P1	110.19 (13)	H9—O1W—H11	112.2
N1—C1—P2	107.45 (12)	H10—O1W—H11	102.3
C2—C1—P2	109.40 (13)	H9—O1W—H12	120.3
P1—C1—P2	114.79 (9)	H10—O1W—H12	98.6
C1—C2—H2A	109.5	H11—O1W—H12	107.6

### Hydrogen-bond geometry (Å, °)

**Table:**

*D*—H···*A*	*D*—H	H···*A*	*D*···*A*	*D*—H···*A*
N1—H1A···O3^i^	0.89	2.02	2.798 (2)	145
N1—H1B···O2^ii^	0.89	2.00	2.766 (2)	144
N1—H1C···O6^i^	0.89	1.93	2.771 (2)	156
O1—H1···O3^iii^	0.82	1.69	2.501 (2)	168
O4—H4···O2^iv^	0.82	1.84	2.635 (2)	164
O1W—H9···O1^v^	0.89	2.06	2.923 (2)	164
O1W—H10···O5	0.89	1.88	2.737 (2)	164
O1W—H11···O6^i^	0.88	1.94	2.781 (2)	159
O1W—H12···O5^vi^	0.89	2.01	2.901 (3)	180

Symmetry codes: (i) *x*+1, *y*, *z*; (ii) −*x*+2, −*y*+2, −*z*+1; (iii) −*x*+1, −*y*+1, −*z*+1; (iv) −*x*+1, −*y*+2, −*z*+1; (v) *x*, *y*, *z*−1; (vi) −*x*+2, −*y*+2, −*z*.
